# “*Candidatus* Ethanoperedens,” a Thermophilic Genus of *Archaea* Mediating the Anaerobic Oxidation of Ethane

**DOI:** 10.1128/mBio.00600-20

**Published:** 2020-04-21

**Authors:** Cedric Jasper Hahn, Rafael Laso-Pérez, Francesca Vulcano, Konstantinos-Marios Vaziourakis, Runar Stokke, Ida Helene Steen, Andreas Teske, Antje Boetius, Manuel Liebeke, Rudolf Amann, Katrin Knittel, Gunter Wegener

**Affiliations:** a Max-Planck Institute for Marine Microbiology, Bremen, Germany; b MARUM, Center for Marine Environmental Sciences, University of Bremen, Bremen, Germany; c Alfred Wegener Institute Helmholtz Center for Polar and Marine Research, Bremerhaven, Germany; d K.G. Jebsen Centre for Deep Sea Research and Department of Biological Sciences, University of Bergen, Bergen, Norway; e University of Patras, Patras, Greece; f The University of North Carolina at Chapel Hill, Chapel Hill, North Carolina, USA; University of Southern California

**Keywords:** alkane degradation, archaea, syntrophy, methyl-coenzyme M reductase, model organism, hydrothermal vents

## Abstract

Cold seeps and hydrothermal vents deliver large amounts of methane and other gaseous alkanes into marine surface sediments. Consortia of archaea and partner bacteria thrive on the oxidation of these alkanes and its coupling to sulfate reduction. The inherently slow growth of the involved organisms and the lack of pure cultures have impeded the understanding of the molecular mechanisms of archaeal alkane degradation. Here, using hydrothermal sediments of the Guaymas Basin (Gulf of California) and ethane as the substrate, we cultured microbial consortia of a novel anaerobic ethane oxidizer, “*Candidatus* Ethanoperedens thermophilum” (GoM-Arc1 clade), and its partner bacterium “*Candidatus* Desulfofervidus auxilii,” previously known from methane-oxidizing consortia. The sulfate reduction activity of the culture doubled within one week, indicating a much faster growth than in any other alkane-oxidizing archaea described before. The dominance of a single archaeal phylotype in this culture allowed retrieval of a closed genome of “*Ca.* Ethanoperedens,” a sister genus of the recently reported ethane oxidizer “*Candidatus* Argoarchaeum.” The metagenome-assembled genome of “*Ca.* Ethanoperedens” encoded a complete methanogenesis pathway including a methyl-coenzyme M reductase (MCR) that is highly divergent from those of methanogens and methanotrophs. Combined substrate and metabolite analysis showed ethane as the sole growth substrate and production of ethyl-coenzyme M as the activation product. Stable isotope probing demonstrated that the enzymatic mechanism of ethane oxidation in “*Ca.* Ethanoperedens” is fully reversible; thus, its enzymatic machinery has potential for the biotechnological development of microbial ethane production from carbon dioxide.

## Abstract (precis)

In the seabed, gaseous alkanes are oxidized by syntrophic microbial consortia that thereby reduce fluxes of these compounds into the water column. Because of the immense quantities of seabed alkane fluxes, these consortia are key catalysts of the global carbon cycle. Due to their obligate syntrophic lifestyle, the physiology of alkane-degrading archaea remains poorly understood. We have now cultivated a thermophilic, relatively fast-growing ethane oxidizer in partnership with a sulfate-reducing bacterium known to aid in methane oxidation and have retrieved the first complete genome of a short-chain alkane-degrading archaeon. This will greatly enhance the understanding of nonmethane alkane activation by noncanonical methyl-coenzyme M reductase enzymes and provide insights into additional metabolic steps and the mechanisms underlying syntrophic partnerships. Ultimately, this knowledge could lead to the biotechnological development of alkanogenic microorganisms to support the carbon neutrality of industrial processes.

## INTRODUCTION

In deep marine sediments, organic matter undergoes thermocatalytic decay, resulting in the formation of natural gas (methane to butane) and crude oil. If not capped, the gas fraction will rise toward the sediment surface due to buoyancy, porewater discharge, and diffusion. Most of the gas is oxidized within the sediments coupled to the reduction of the abundant electron acceptor sulfate (1, 2). Responsible for the anaerobic oxidation of alkanes are either free-living bacteria or microbial consortia of archaea and bacteria. Most free-living bacteria use alkyl succinate synthases to activate the alkane, forming succinate-bound alkyl units as primary intermediates (3). Usually, these alkanes are completely oxidized, and this process is coupled to sulfate reduction in the same cells, as has been shown, for example, in the deltaproteobacterial butane-degrading strain BuS5 (4). However, alkane oxidation in seafloor sediments is to a large extent performed by dual species consortia of archaea and bacteria (5, 6). As close relatives of methanogens, the archaea in these consortia activate alkanes as thioethers and completely oxidize the substrates to CO_2_. The electrons released during alkane oxidation are consumed by the sulfate-reducing partner bacteria.

The anaerobic methane-oxidizing archaea (ANME) activate methane using methyl-coenzyme M (CoM) reductases (MCRs) that are highly similar to those of methanogens, forming methyl-coenzyme M as the primary intermediate (7). The methyl group is oxidized via a reversal of the methanogenesis pathway (8). Thermophilic archaea of the genus “*Candidatus* Syntrophoarchaeum” thrive on the oxidation of butane and propane. In contrast to ANME, they contain four highly divergent MCR variants, which generate butyl- and propyl-coenzyme M (CoM) as primary intermediates (9). Based on genomic and transcriptomic evidence, the CoM-bound alkyl units are transformed to fatty acids and oxidized further via beta-oxidation. The reactions transforming the CoM-bound alkyl units to CoA-bound fatty acids and the enzymes performing such reactions are so far unknown. The CoA-bound acetyl units are completely oxidized in the Wood-Ljungdahl pathway including the upstream part of the methanogenesis pathway. In hydrogenotrophic methanogens, the enzymes of this pathway are used to reduce CO_2_-forming methyl-tetrahydromethanopterin for methanogenesis and for biomass production. In “*Ca.* Syntrophoarchaeum,” this pathway is used in reverse direction for the complete oxidation of acetyl-CoA. Both the thermophilic ANME-1 and “*Ca*. Syntrophoarchaeum” form dense consortia with their sulfate-reducing partner bacterium “*Candidatus* Desulfofervidus” (HotSeep-1 clade) (10, 11). The transfer of reducing equivalents between the alkane-oxidizing archaea and their partners is likely mediated by pilus-based nanowires and cytochromes produced by the two consortial partners (12). For a critical view on electron transfer in anaerobic oxidation of methane (AOM) consortia, see reference 13.

Sulfate-dependent ethane oxidation has been described multiple times in slurries of marine sediments (4, 14, 15). The first functional description of this process was based on a cold-adapted culture derived from Gulf of Mexico sediments (5). In this culture, “*Candidatus* Argoarchaeum” (formerly known as GoM-Arc1 clade) activates ethane with the help of divergent MCRs that are phylogenetically placed on a distinct branch next to those of “*Ca.* Syntrophoarchaeum.” Based on the presence of all enzymes of the Wood-Ljungdahl pathway that can be used for acetyl-CoA oxidation, it has been suggested that the CoM-bound ethyl groups are transferred to CoA-bound acetyl units. The required intermediates for this reaction mechanism are so far unknown (5). “*Ca.* Argoarchaeum” forms unstructured consortia with yet-unidentified bacterial partners and grows slowly with substrate turnover rates comparable to AOM (5). Additional metagenome-assembled genomes (MAGs) of the GoM-Arc1 clade derived from the Guaymas Basin and the Gulf of Mexico have similar gene contents, suggesting that these GoM-Arc1 archaea are ethane oxidizers (16, 17).

To date, the understanding of short-chain alkane-metabolizing archaea mainly relies on comparison of their genomic information with those of methanogens that are well characterized with regard to their enzymes. Due to the slow growth of the alkane-oxidizing archaea and the resulting lack of sufficient biomass, specific biochemical traits remain unknown. For instance, the structural modifications of noncanonical MCRs or the proposed transformation of the CoM-bound alkyl to CoA-bound acetyl units in the short-chain alkane degraders has not been proven. Here, we describe a faster-growing, thermophilic ethane-oxidizing culture from sediments of the Guaymas Basin. Metagenomic analyses of Guaymas Basin sediments revealed a great diversity of potential alkane degraders with divergent MCR enzymes (9, 18). With ethane as sole energy source and sulfate as electron acceptor, we obtained well-growing meso- and thermophilic ethane-degrading enrichment cultures from these sediments. Their low strain diversity makes them particularly suitable for assessing the pathways of the anaerobic oxidation of ethane.

## 

### Taxonomy of “*Candidatus* Ethanoperedens thermophilum.”

Etymology: *ethano* (new Latin), pertaining to ethane; *peredens* (Latin), consuming, devouring; *thermophilum* (Greek), heat-loving. The name implies an organism capable of ethane oxidation at elevated temperatures. Locality: enriched from hydrothermally heated, hydrocarbon-rich marine sediment of the Guaymas Basin at 2,000-m water depth, Gulf of California, Mexico. Description: anaerobic, ethane-oxidizing archaeon, mostly coccoid, about 0.7 μm in diameter, forms large irregular cluster in large dual-species consortia with the sulfate-reducing partner bacterium “*Candidatus* Desulfofervidus auxilii.”

## RESULTS AND DISCUSSION

### Establishment of meso- and thermophilic ethane-oxidizing enrichment cultures.

**FIG 1:**
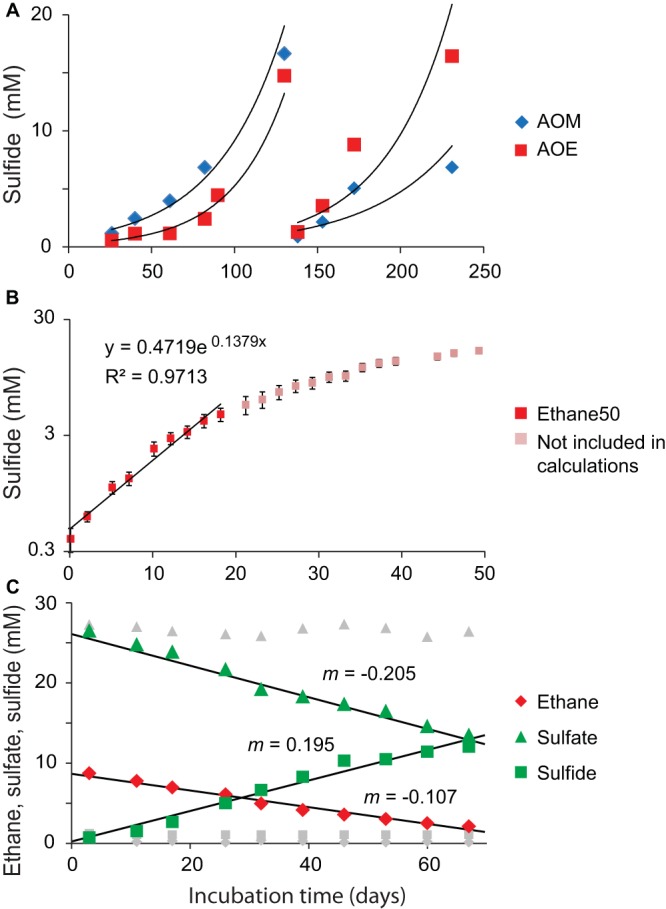
Cultivation and stoichiometry test of the Ethane50 culture. (A) Rates of methane-dependent (blue) and ethane-dependent (red) sulfide production in sediments of the Guaymas Basin incubated at 50°C. (B) Determination of activity doubling times in anaerobic ethane-oxidizing culture. Logarithmic *y* axis with sulfide production shows a decrease in activity at 3 mM sulfide and estimated activity doubling times in low sulfide concentrations of 6 to 7 days. (C) Development of ethane (diamonds), sulfate (triangles), and sulfide (squares) concentrations in the Ethane50 culture. Gray symbols show corresponding concentrations measured in control incubations without ethane addition (data from 1 of 3 replicate incubations; for complete data, see Table S6). The ratios of the slopes of sulfate and sulfide to ethane (1.92 and 1.82, respectively) are close to the stoichiometric ratios of sulfate reduction and ethane oxidation. The small offset may relate to biomass production and sampling artifacts.

Sediments were sampled from the gas- and oil-rich sediments covered by sulfur-oxidizing mats of the Guaymas Basin. From these sediments and artificial seawater medium, a slurry was produced under anoxic conditions and distributed into replicate bottles. These bottles were supplied with an ethane headspace (2 atm) and incubated at 37°C and 50°C. Additional growth experiments were performed with methane, and controls were set up with a nitrogen atmosphere. As a measure of metabolic activity, sulfide concentrations were tracked over time (for further details, see Materials and Methods). Both methane and ethane additions resulted in the formation of 15 mM sulfide within 4 months. Nitrogen controls produced only little sulfide (<2 mM) that likely corresponds to the degradation of alkanes and organic matter from the original sediment. Subsequent dilution (1:3) of the ethane and methane cultures and further incubation with the corresponding substrates showed faster, exponentially increasing sulfide production in the ethane culture, suggesting robust growth of the ethane-degrading community (Fig. 1A). After three consecutive dilution steps, virtually sediment-free cultures were obtained. These cultures produced approximately 10 mM sulfide in 8 weeks. All further experiments were conducted with the faster-growing 50°C culture (Ethane50). Sequencing of metagenomes, however, was done on both, the 50°C and 37°C (Ethane37) culture.

A stoichiometric growth experiment with the Ethane50 culture (Fig. 1B) showed that ethane was completely oxidized while sulfate was reduced to sulfide according to the formula 4C_2_H_6_ + 7SO_4_^2−^ → 8HCO_3_^−^ + 7HS^−^ + 4H_2_O + H^+^.

An experiment tracking the exponential development of sulfide over time suggested doubling times of only 6 days at low sulfide concentrations of <5 mM (Fig. 1B), which is substantially faster than estimated for thermophilic AOM consortia, with about 60 days (10), and also faster than the cold-adapted anaerobic ethane-oxidizing cultures (5). Sulfide concentrations over 5 mM seemed to suppress activity and growth of the ethane-oxidizing microorganisms (Fig. 1C). Hence, flowthrough bioreactors could be beneficial to increase biomass yields of anaerobic ethane degraders.

### Microbial composition of the Ethane50 culture.

**FIG 2:**
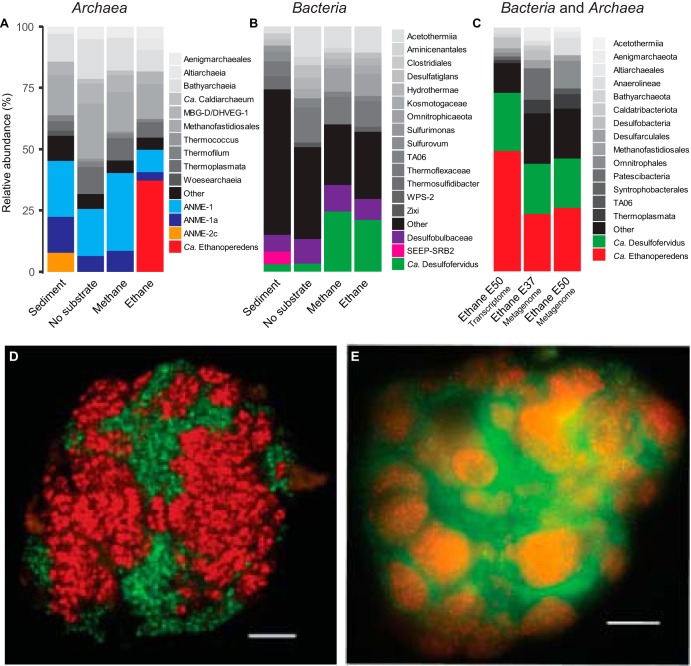
Microbial composition of the Ethane50 culture. (A and B) Relative abundance of phylogenetic clades of archaea (A) and bacteria (B) based on 16S rRNA gene amplicon sequencing present in the inoculated sediment, and in cultures with no substrate, with methane and ethane after 150 days of incubation. (C) Relative abundance of active microbial groups based on 16 rRNA fragments recruited from the genome of Ethane37 and Ethane50 after 2.5 years of incubation and the transcriptome of the Ethane50 culture after 1 year of incubation with ethane. (D and E) Laser-scanning micrograph (D) and epifluorescence micrograph (E) of microbial consortia stained with probes specific for the GoM-Arc1 clade (red, Alexa 594) and **“***Ca.* Desulfofervidus” (green, Alexa 488) in the Ethane50 culture. Bar, 10 μm.

Amplified archaeal and bacterial 16S rRNA genes of the original sediment and early, still sediment-containing cultures (150 days of incubation) were sequenced to track the development of microbial compositions over time (for primers, see Table S1 in the supplemental material). The original sediment contained large numbers of ANME-1 and the putative partner bacterium “*Ca.* Desulfofervidus.” The AOM culture became further enriched in ANME-1 archaea and “*Ca.* Desulfofervidus,” whereas in the Ethane50 culture the GoM-Arc1 clade increased from <0.1% in the original sediment to roughly 35% of all archaea (Fig. 2A). Notably, the relative abundance of “*Ca.* Desulfofervidus” increased also in the Ethane50 culture. This indicates that “*Ca.* Desulfofervidus” was also involved as a partner bacterium in the thermophilic ethane culture.

10.1128/mBio.00600-20.5 TABLE S1 PCR primers used in the amplification of archaeal and bacterial 16S rRNA genes and oligonucleotide probes used for CARD-FISH. Download Table S1, XLSX file, 0.01 MB. Copyright © 2020 Hahn et al. 2020 Hahn et al. This content is distributed under the terms of the Creative Commons Attribution 4.0 International license.

To visualize the cells involved in the anaerobic oxidation of ethane (AOE), oligonucleotide probes specific for the GoM-Arc1 clade and “*Ca.* Desulfofervidus” were applied on the Ethane50 culture using catalyzed reporter deposition fluorescence *in situ* hybridization (CARD-FISH; for probes, see Table S1). The Ethane50 culture contained large and tightly packed consortia with sizes of up to 40 μm in diameter formed by GoM-Arc1 and “*Ca.* Desulfofervidus” cells (Fig. 2D and E). In the consortia, archaea and bacteria grew spatially separated. These large consortia apparently develop from small but already dense consortia found in the inoculate, similar to what was found for cold-adapted AOM consortia (19). Such a separation of the partner organisms is also characteristic for consortia in the butane-degrading culture (9) and for most AOM consortia (20). In contrast, in thermophilic AOM consortia of ANME-1 and “*Ca.* Desulfofervidus,” the partner cells appear well mixed (21). The Ethane50 culture differs from the cold-adapted ethane-oxidizing culture, in which “*Ca.* Argoarchaeum” forms rather loose assemblages with yet-uncharacterized bacteria (5).

To analyze the metabolic potential of the microorganisms involved in ethane degradation, Ethane37 and Ethane50 cultures were subjected to transcriptomic and genomic analysis. The 16S rRNA sequences extracted from the shotgun RNA reads of the Ethane50 culture were strongly dominated by GoM-Arc1 (50%) and “*Ca.* Desulfofervidus” (20%; Fig. 2C), supporting a crucial role of these two organisms in thermophilic ethane degradation. Long-read DNA sequencing for the Ethane50 culture resulted in a partial genome of GoM-Arc1 with 76.2% completeness (GoM-Arc1_E50_DN), whereas by applying this approach to the Ethane37 culture, we obtained a closed genome of the GoM-Arc1 archaeon (GoM-Arc1_E37). The two GoM-Arc1 genomes share an average nucleotide identity (ANI) of 98%; hence, a complete consensus genome for Ethane50 (GoM-Arc1_E50) was obtained by mapping long reads of the Ethane50 culture on the closed GoM-Arc1_E37 genome (see Materials and Methods and Table S2). GoM-Arc1_E50 had a size of 1.92 Mb and a GC content of 46.5%. To assess the genomic diversity of archaea of the GoM-Arc1 clade, additionally a MAG of GoM-Arc1 from the Loki’s Castle hydrothermal vent field (GoM-Arc1-LC), with a completeness of 68% and eight single-cell amplified genomes (SAGs) from different cold seeps and different completenesses (10% to 59%) were retrieved (Table S2). The MAG GoM-Arc1-LC and the eight single cells have an average nucleotide identity (ANI) of over 90%, suggesting that they belong to the same or closely related species. The 16S rRNA gene identity is in the range of 99.5%, supporting a definition as same species, and shows that the same species of GoM-Arc1 can be found in diverse seep sites (Table S2 and Fig. S1). Together with several MAGs of the GoM-Arc1 clade archaea from public databases (5, 17, 18) these MAGs now provide an extensive database for the genomic description of the GoM-Arc1 clade. All GoM-Arc1 clade genomes have an estimated size smaller than 2 Mb, which is in the range of the other thermophilic alkane degraders, such as “*Ca.* Syntrophoarchaeum” (1.5 to 1.7 Mb) and ANME-1 (1.4 to 1.8 Mb) (9, 22). The genome is, however, much smaller than the 3.5-Mb genome of the mesophilic sister lineage “*Candidatus* Methanoperedens.” This organism thrives on methane and is able to reduce nitrate or metals without partner bacteria (23, 24).

10.1128/mBio.00600-20.1 FIG S1 Phylogenetic affiliation of the GoM-Arc1 clade archaea with other archaea based on 16S rRNA gene comparison. The tree was constructed using ARB (56) and the FastTree 2 package (57) using a 50% similarity filter. Four hundred ten sequences with a length of at least 1,100 bp, excluding partial sequences retrieved from single cells, were used. Bar shows 10% sequence divergence. Download FIG S1, EPS file, 1.0 MB. Copyright © 2020 Hahn et al. 2020 Hahn et al. This content is distributed under the terms of the Creative Commons Attribution 4.0 International license.

10.1128/mBio.00600-20.6 TABLE S2 Summary of single-cell and metagenome-assembled genomes presented in this study and average nucleotide and amino acid identities. ANI and AAI values were calculated with publicly available genomes and genomes presented in this study. Enveomics tools were used for the calculation (50). Download Table S2, XLSX file, 0.03 MB. Copyright © 2020 Hahn et al. 2020 Hahn et al. This content is distributed under the terms of the Creative Commons Attribution 4.0 International license.

**FIG 3:**
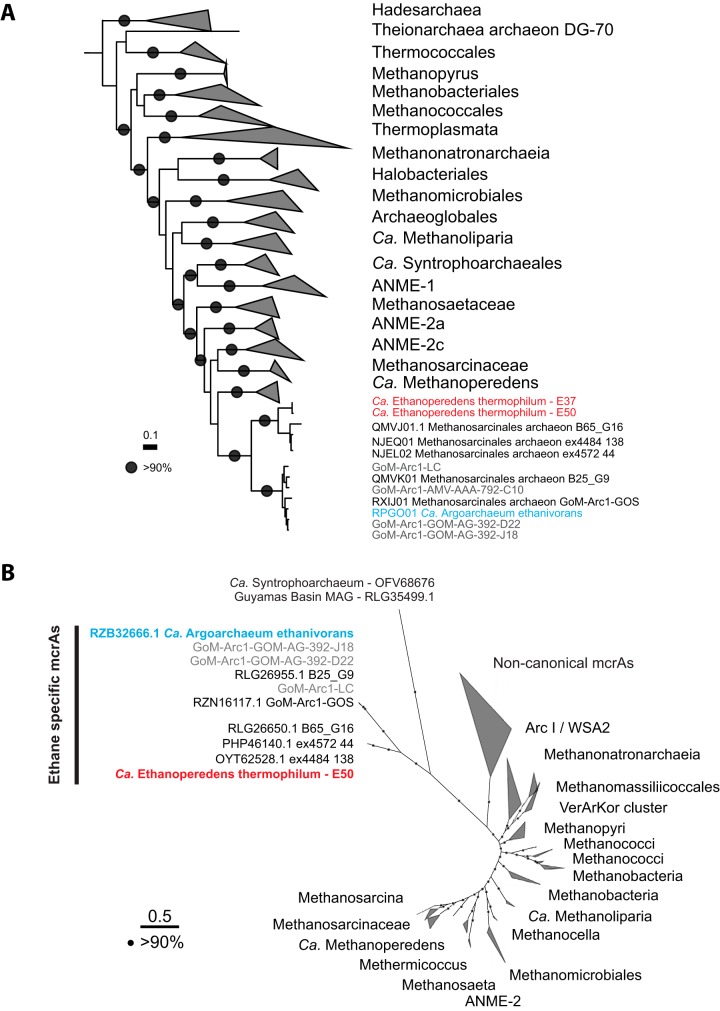
Phylogenetic affiliation based on 32 marker genes and *mcr*A amino acid sequences of “*Ca.* Ethanoperedens.” (A) Phylogenetic affiliation of “*Ca.* Ethanoperedens” within the *Euryarchaeota* based on 32 aligned marker gene amino acid sequences; outgroup is *Thaumarchaeota*. The scale bar indicates 10% sequence divergence. (B) Phylogenetic affiliation of *mcr*A amino acid sequences. The *mcr*A sequences of GoM-Arc1 form a distinct branch within the noncanonical, potentially multicarbon alkane-activating MCRs. The *mcr*A genes of the GoM-Arc1 cluster can be further divided into those from cold-adapted organisms, including “*Ca.* Argoarchaeum ethanivorans,” and the cluster including the thermophiles of the genus “*Ca.* Ethanoperedens.” Sequences from the Ethane50 enrichment are depicted in red, environmental sequences from metagenomes and single-cell genomes from this study are in gray, and “*Ca.* Argoarchaeum ethanivorans” sequences are in blue. The VerArKor cluster contains *mcr*A sequences belonging to the Verstraetearchaeota, Archaeoglobus, and Korarchaeota.

All GoM-Arc1 genomes contain the genes encoding the enzymes of the methanogenesis pathway, including a highly similar divergent-type MCR and the Wood-Ljungdahl pathway, but no pathway for beta-oxidation of longer fatty acids. Hence, it is likely that all members of this clade are ethane oxidizers. Based on 16S rRNA gene phylogeny and a genome tree based on 32 marker genes, the GoM-Arc1 clade divides into two subclusters. According to a 16S rRNA gene identity of ∼95% (Fig. S1) and an average amino acid identity (AAI) of ∼63% (Fig. 3A; Table S2), these clusters should represent two different genera. One cluster contains the recently described ethane oxidizer “*Candidatus* Argoarchaeum ethanivorans” and genomes derived from cold environments including the Gulf of Mexico and the moderately heated Loki’s Castle seeps (25). The second cluster includes the thermophilic GoM-Arc1 strains found in the Ethane50 and Ethane37 cultures and sequences of other MAGs from the Guaymas Basin (16, 18). Based on the substrate specificity (see results below) and its optimal growth at elevated temperatures, we propose to name the Ethane50 strain of GoM-Arc1 “*Candidatus* Ethanoperedens thermophilum” (*Ethanoperedens*, Latin for nourishing on ethane; *thermophilum*, Latin for heat loving).

### Genomic and catabolic features of “*Ca.* Ethanoperedens.”

**FIG 4:**
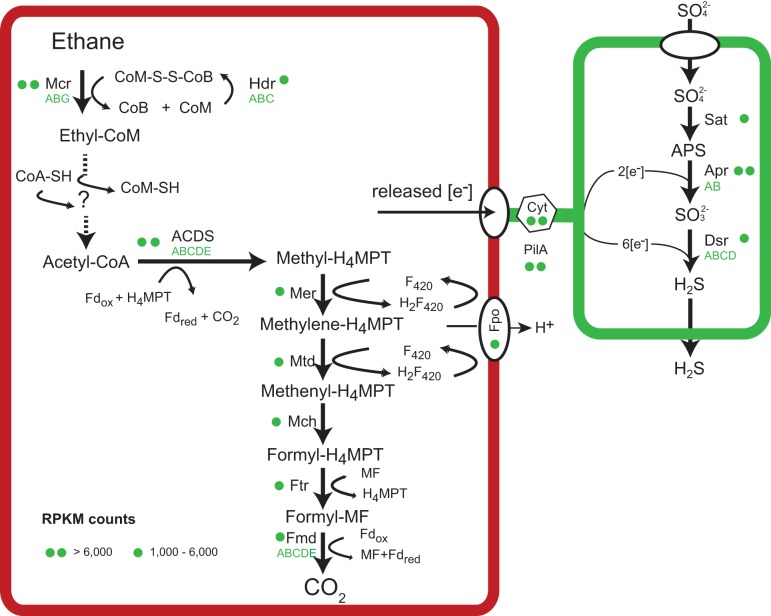
Metabolic model of anaerobic ethane oxidation in “*Ca.* Ethanoperedens thermophilum.” Ethane is activated in the ethane-specific MCR. The produced CoM-bound ethyl groups are consecutively oxidized and transformed to CoA-bound acetyl units. Acetyl-CoA is cleaved using the ACDS of the Wood-Ljungdahl pathway. The remaining methyl groups are fully oxidized on the reversed methanogenesis pathway. Similarly to ANME archaea and **“***Ca.* Syntrophoarchaeum,” “*Ca.* Ethanoperedens” does not contain a reductive pathway; hence, electrons released during ethane oxidation are transferred to the partner bacterium “*Ca.* Desulfofervidus auxilii.” Therefore, in both partners, cytochromes and pili are present and expressed, similarly to what is described in thermophilic consortia performing AOM (22) (for detailed expression patterns, see Table S3).

The main catabolic pathways of “*Ca.* Ethanoperedens” are a complete methanogenesis and a Wood-Ljungdahl pathway (Fig. 4). Its genome encodes only one MCR. The three MCR subunits αβγ are on a single operon. The amino acid sequence of the alpha subunit (*mcr*A) of “*Ca.* Ethanoperedens” is phylogenetically most closely related to the recently described divergent-type MCR of “*Ca.* Argoarchaeum” with an amino acid identity of 69% but also with all other *mcr*A sequences of GoM-Arc1 archaea (5, 12, 16, 18). These MCRs form a distinct cluster in comparison to other divergent MCRs and to the canonical MCRs of methanogens and methanotrophs (Fig. 3B). The similarity of GoM-Arc1 *mcr*A sequences to the described canonical and noncanonical sequences is below 43%, and changes in the amino acid sequences are also found in the highly conserved active site of the enzyme (Fig. S2). The relative expression of the *mcr* subunits compared to all reads mapping to “*Ca.* Ethanoperedens” (reads per kilobase per million mapped reads [RPKM], i.e., *mcr*A = 9,790) is at least two times higher than the expression of all other genes of the main catabolic pathway (Fig. 4; Table S3). The relative *mcr* expression of “*Ca.* Ethanoperedens” is higher than the expression of the multiple *mcr* genes in “*Ca.* Syntrophoarchaeum” but lower than the expression of *mcr* in thermophilic ANME-1 archaea (9, 22). The relatively low expression of *mcr* in short-chain alkane-oxidizing archaea can be explained by the properties of their substrates. Short-chain alkane oxidation releases larger amounts of energy than methane oxidation. Furthermore, the cleavage of C-H bonds in multicarbon compounds requires less energy than the cleavage of C-H bonds of methane (26); hence, less MCR might be required to supply the organism with sufficient energy.

10.1128/mBio.00600-20.2 FIG S2 Comparison of *mcr*A sequences from the GoM-Arc1 clade to described canonical and noncanonical *mcr*A sequences. (A) Alignment of the active site of the *mcr*A genes from different representative genomes. The four different “*Ca.* Syntrophoarchaeum” sequences belong to the same genome bin. Amino acid positions refer to “*Ca.* Ethanoperedens thermophilum” Ethane50 *mcr*A sequence. (B) Identity matrix of *mcr*A sequences based on NCBI blastp alignment. Download FIG S2, EPS file, 1.2 MB. Copyright © 2020 Hahn et al. 2020 Hahn et al. This content is distributed under the terms of the Creative Commons Attribution 4.0 International license.

10.1128/mBio.00600-20.7 TABLE S3 Genomes and gene expression data of the Ethane50 culture and overview of genes potentially involved in the ethane metabolism and electron cycling in the Ethane50 culture. Expression values are shown in triplicates for “*Ca.* Ethanoperedens thermophilum” and “*Ca.* Desulfofervidus auxilii.” Download Table S3, XLSX file, 0.4 MB. Copyright © 2020 Hahn et al. 2020 Hahn et al. This content is distributed under the terms of the Creative Commons Attribution 4.0 International license.

**FIG 5:**
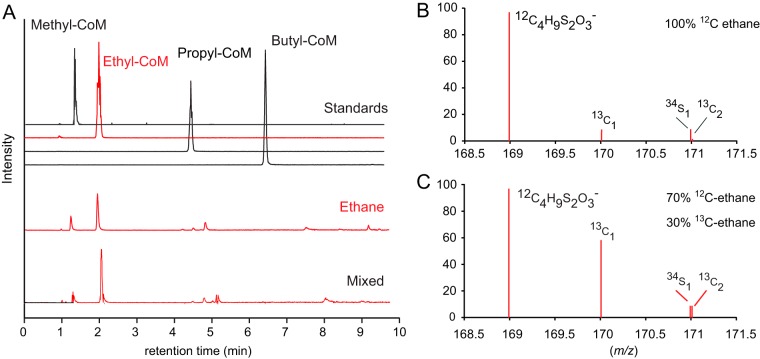
Detection of coenzyme M-bound intermediates in the Ethane50 culture. (A) Top four lines show total ion counts for UHPLC peaks for authentic standards of methyl-, ethyl-, propyl-, and butyl-CoM, respectively, and chromatograms for ethane and mixed alkane gases (methane to butane). (B and C) Mass spectra (*m/z* 168.5 to 171.5) for culture extracts after providing the Ethane50 culture with nonlabeled ethane (B) and 30% ^13^C-labeled ethane (C). Diagrams show the relative intensities (*y* axes) for ethyl-CoM-H (^12^C_4_H_9_S_2_O_3_^−^) (calculated *m/z* 168.9988) and its isotopologues with [1-^13^C]ethyl-CoM or [2-^13^C]ethyl-CoM or one ^34^S isotope.

To test the substrates activated by the MCR of “*Ca.* Ethanoperedens,” we supplied different alkanes to the active Ethane50 culture replicates and analyzed the extracted metabolites. Cultures supplied with ethane show the *m/z* 168.9988 of the authentic ethyl-CoM standard (Fig. 5A and B), which was not observed in the control incubation without substrate. Moreover, addition of 30% [1-^13^C]ethane resulted in the increase of masses expected for [1-^13^C]ethyl-CoM and [2-^13^C]ethyl-CoM (Fig. 5C). This confirms that “*Ca.* Ethanoperedens” produces ethyl-CoM from ethane. To test substrate specificity of “*Ca.* Ethanoperedens,” we provided culture replicates with four different gaseous alkanes (methane, ethane, propane, and *n*-butane and a mix of all four substrates). Besides the ethane-amended culture, sulfide was produced only in the Ethane50 culture supplied with the substrate mix (Fig. S3). In agreement with this, no other alkyl-CoM variant apart from ethyl-CoM was detected (Fig. 5A). This shows that the MCR of “*Ca.* Ethanoperedens” and most likely all MCR enzymes of GoM-Arc1 archaea (Fig. 3B) activate ethane but no or only trace amounts of methane and other alkanes. The high substrate specificity of the MCR is crucial for GoM-Arc1 archaea, since they lack the fatty acid degradation pathway that is required to degrade butane and propane (9). “*Ca.* Ethanoperedens” contains and expresses a complete methyltransferase (*mtr*). The corresponding enzyme might cleave small amounts of methyl-CoM that might be formed as a side reaction of the MCR. The methyl unit would be directly transferred to the methylene-tetrahydromethanopterin (H_4_-MPT) reductase (*mer*) and oxidized in the upstream part of the methanogenesis pathway to CO_2_ (Fig. 4).

10.1128/mBio.00600-20.3 FIG S3 Development of sulfide concentrations in substrate experiments in replicate incubations of the Ethane50 culture supplied with ethane, alternative alkanes, or a mix of these substrates. Results show that ethane is the only alkane used as electron donor in “*Ca*. Ethanoperedens thermophilum.” Download FIG S3, EPS file, 0.4 MB. Copyright © 2020 Hahn et al. 2020 Hahn et al. This content is distributed under the terms of the Creative Commons Attribution 4.0 International license.

Based on the observed net reaction and the genomic information, “*Ca.* Ethanoperedens” completely oxidizes ethane to CO_2_. In this pathway, coenzyme A-bound acetyl units are oxidized in the Wood-Ljungdahl pathway including the upstream part of the methanogenesis pathway (Fig. 4). Our model, however, does not explain how CoM-bound ethyl groups are oxidized to acetyl units and ligated to CoA. Similar transformations are required in the other multicarbon alkane-oxidizing archaea, such as “*Ca.* Syntrophoarchaeum” and “*Ca.* Argoarchaeum” (5, 9). Those oxidation reactions lack biochemical analogues; hence, genomic information alone allows only indirect hints on their function. In “*Ca.* Ethanoperedens,” a release of ethyl units and transformation as free molecules (ethanol to acetate) is unlikely, because a formation of acetyl-CoA from acetate would require CoA ligases, which are not present in the genome. Instead, the transformation of ethyl into acetyl units could be performed by a tungstate-containing aldehyde ferredoxin oxidoreductase (AOR) that could catalyze the oxidation with cofactors such as CoM or CoA. In the archaeon Pyrococcus furiosus, AORs transform aldehydes to the corresponding carboxylic acid (27). Both “*Ca*. Ethanoperedens” and “*Ca.* Argoarchaeum” genomes contain three *aor* copies, and in all cases these genes are located either in close proximity to or on operons with genes of the methanogenesis pathway. We detected a high expression of two of the three *aor* genes (RPKM *aor *= 3,805 and 7,928), indicating a viable function of the enzymes. Likewise, very high protein concentrations of these enzymes were shown for “*Ca*. Argoarchaeum” (5), supporting the hypothesis of a critical function. An *aor* gene is also present in the butane oxidizer “*Ca.* Syntrophoarchaeum,” yet its expression is rather moderate (9), which puts in question its role in the catabolic pathway of this organism. In contrast, ANME archaea do not contain or overexpress *aor* genes, likely because the encoded enzymes have no central role in their metabolism. We searched the cell extracts for potential intermediates in the pathway, but based on retention time and mass, we were not able to detect potential intermediates such as ethyl-CoA. Similarly, acetyl-CoA, the substrate of the Wood-Ljungdahl pathway, was not detected. A lack of detection, however, does not exclude those compounds as intermediates. Instead, the compound turnover might be very fast, which could be required for an efficient net reaction. Additionally, a mass spectrometric detection of unknown intermediates could be hindered by compound instability or loss during the extraction. Further metabolite studies and enzyme characterizations are required to understand the role of AOR in alkane oxidation

Acetyl-CoA, the product formed by the above-proposed reactions, can be introduced into the Wood-Ljungdahl pathway. The acetyl group is decarboxylated by the highly expressed acetyl-CoA decarbonylase/synthase (ACDS), and the remaining methyl group is transferred to tetrahydromethanopterin (H_4_-MPT). The formed methyl-H_4_-MPT can then be further oxidized to CO_2_ following the reverse methanogenesis pathway (Fig. 4). “*Ca.* Ethanoperedens” lacks genes for sulfate or nitrate reduction, similarly to other genomes of the GoM-Arc1 clade. The electrons produced in the oxidation of ethane thus need to be transferred to the sulfate-reducing partner bacterium “*Ca*. Desulfofervidus auxilii,” as previously shown for the anaerobic oxidation of methane and butane. In cocultures of “*Ca.* Argoarchaeum” and their partner bacteria, Chen and coworkers (5) suggest the transfer of reducing equivalents via zero-valent sulfur between the loosely aggregated “*Ca*. Argoarchaeum” and its partner bacterium, analogous to the hypothesis of Milucka et al. (28). In the Ethane50 culture, such a mode of interaction is highly unlikely, as the partner “*Ca*. Desulfofervidus auxilii” is an obligate sulfate reducer, incapable of sulfur disproportionation (11). Based on genomic information, direct electron transfer appears to be more likely. Alkane-oxidizing archaea and their partner bacterium “*Ca.* Desulfofervidus auxilii,” produce cytochromes and pilus-based nanowires when supplied with their substrate (9, 29, 30). Also, “*Ca.* Ethanoperedens” contains 11 different genes for cytochromes with expression values of up to 14,800 RPKM representing some of the highest-expressed genes in the culture (Table S3). Interestingly, “*Ca.* Ethanoperedens” also contains and expresses a type IV pilin protein with a high RPKM value of 11,246. The partner bacterium “*Ca.* Desulfofervidus” also shows a high expression of pili and cytochromes under ethane supply, showing their potential importance for the interaction of these two organisms in the syntrophic coupling of ethane oxidation to sulfate reduction.

### Environmental distribution of GoM-Arc1 archaea.

**FIG 6:**
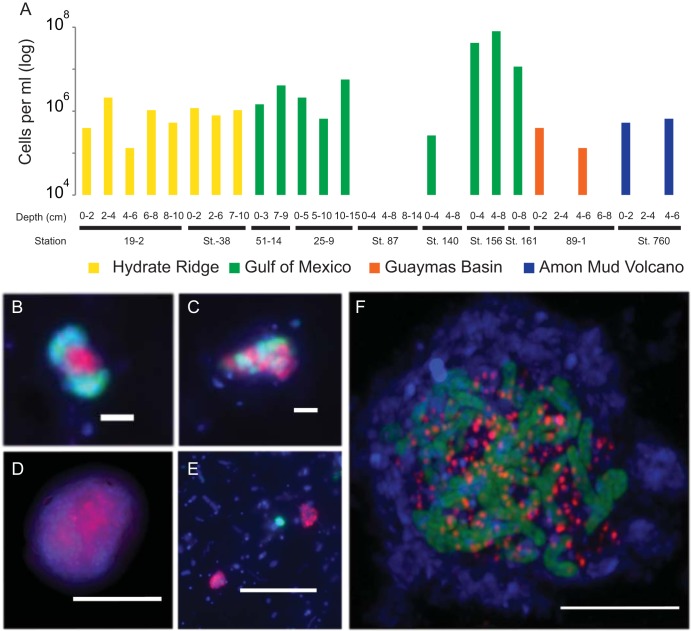
Abundance and exemplary micrographs of GoM-Arc1 archaea in sediments from cold seeps and Guaymas Basin. (A) Abundance estimations of archaeal cells detected by the GoM-Arc1-specific probe GOM-ARCI-660 in a CARD-FISH survey. Detection limit, approximately 5 × 10^4^ cells per ml sediment. (B to F) Epifluorescence (B to E) and laser scanning (F) micrographs of environmental samples using CARD-FISH with combination of the GoM-Arc1-specific probe (red) and the general bacterial probe EUB-338 (green). Environmental samples originated from the seep sites Hydrate Ridge, Oregon (B); Gulf of Mexico (C); Guaymas Basin (D); Loki’s Castle (E); and Katakolo Bay, Greece (F). Bars, 5 μm (D to F) and 2 μm (B and C).

16S rRNA gene sequences clustering with “*Ca.* Ethanoperedens” and “*Ca.* Argoarchaeum” have been found in hydrocarbon-rich marine environments like cold-seep and hot-vent environments, including asphalt seeps in the Gulf of Mexico and the Guaymas Basin hydrothermal vents in the Gulf of California (31–33). In some environments like oil seeps of the Gulf of Mexico and gas-rich barite chimneys of Loki’s Castle, 16S rRNA gene surveys have shown that up to 30% of archaeal gene sequences belonged to the GoM-Arc1 clade (12). To estimate absolute abundances and potential partnerships of GoM-Arc1 in the environment, we performed CARD-FISH on samples from different seep and vent sites across the globe (Fig. 6). With up to 10^8^ cells per ml, archaea of the GoM-Arc1 clade were particularly abundant in cold-seep sediments in the northern Gulf of Mexico (station 156). This cold seep transports thermogenic hydrocarbon gases that are particularly enriched in short-chain alkanes (34, 35). Other cold-seep and hot-vent sediments from the Guaymas Basin, Hydrate Ridge, and Amon Mud Volcano contain between 10^5^ and 10^6^ GoM-Arc1 cells per ml of sediment, which represents 1 to 5% of the archaeal community (Fig. 6A). At all sites, we found that GoM-Arc1 associates with partner bacteria. At the hydrothermally heated site in the Guaymas Basin, GoM-Arc1 aggregated with “*Ca.* Desulfofervidus,” the partner bacterium of the Ethane37 and Ethane50 cultures. At Loki’s Castle, GoM-Arc1 and “*Ca.* Desulfofervidus” were cooccurring in barite chimneys based on sequence information, yet they were not found to form the same tight consortia as at other sites. At the temperate site Katakolo Bay in Greece, GoM-Arc1 archaea formed consortia with very large, yet unidentified vibrioform bacteria (Fig. 6B to F). These cells hybridized with a probe for Deltaproteobacteria but not with probes for known partner bacteria (for probes, see Table S1). At the cold-seep sites, the associated cells could not be stained with probes for the known partner bacteria of cold-adapted ANME, including SEEP-SRB1 and SEEP-SRB2, and also not with that for “*Ca.* Desulfofervidus.” It remains an important question as to how the archaea can select only a few specific types of bacteria as partners in the anaerobic alkane oxidation and for which specific traits they are selected. Based on their global presence in hydrocarbon-rich environments, GoM-Arc1 archaea could be considered key players in the anaerobic oxidation of ethane in marine sediments. Their role would be similar to the role of ANME archaea in AOM.

### Future possible applications of “*Ca.* Ethanoperedens.”

Archaea of the GoM-Arc1 cluster are likely the dominant, if not the only, organisms capable of anaerobic oxidation of ethane on the global seafloor. An important further task is to assess deep oil and gas reservoirs for their diversity of ethane oxidizers. The rapid growth of “*Ca.* Ethanoperedens” and the streamlined genome make it a model organism for the study of anaerobic ethanotrophy in archaea. The biochemistry of short-chain alkane-oxidizing archaea will be of high interest for future biotechnological applications. An organism using the metabolism of “*Ca*. Ethanoperedens” in the reverse direction should be able to produce ethane, similarly to methane production by methanogens. Yet, there is scarce isotopic evidence for the existence of ethanogenic organisms in nature (36). Furthermore, under common environmental conditions thermodynamics favor the production of methane from inorganic carbon over the production of ethane. To test the general reversibility of the ethane oxidation pathway, we incubated the active Ethane50 culture with ^13^C-labeled inorganic carbon and traced the label transfer into ethane. Within 18 days, [δ-^13^C]ethane values increased from −3‰ to +120‰, whereas isotopic compositions in the nonlabeled culture remained stable (Fig. S4). Considering the forward rate and ethane stock, the back reaction amounts to 1.5‰ to 3% of the forward reaction, which is in the range for back fluxes of carbon measured in AOM (21, 37). This experiment shows that the ethane oxidation pathway is fully reversible. To test the net ethane formation in the Ethane50 culture, we removed sulfate from culture aliquots and added hydrogen as electron donor. These cultures formed between 1 and 17 μmol liter^−1^ ethane within 27 days (Table S6). The ethane production was, however, a very small fraction (0.08%) of the ethane oxidation rate in replicate incubations with ethane and sulfate. No ethane was formed in the presence of hydrogen and sulfate. We interpret the ethane formation in the culture as enzymatic effect in the ethane-oxidizing consortia. Bacterial hydrogenases will fuel reducing equivalents into the pathway, which may ultimately lead to the reduction of carbon dioxide to ethane. A growing culture could not be established under these conditions, however, the experiments suggest that related or genetically modified methanogenic archaea could thrive as ethanogens. A complete understanding of the pathway and enzymes of GoM-Arc1 archaea, however, is required to develop the biotechnological potential of an ethanogenic organism. To allow energy-conserving electron flows in this organism, a genetically modified methanogen should be used as host organism. For a targeted modification of such archaea, the pathway of ethane oxidation must be completely understood, and research should focus especially on the transformation of coenzyme M-bound ethyl units to coenzyme A-bound acetyl units.

10.1128/mBio.00600-20.4 FIG S4 Test for the transfer of dissolved inorganic carbon into ethane in the Ethane50 culture. (A) Development of sulfide concentrations in the culture with ethane as energy source and sulfate as electron acceptor. (B) Development of δ-^13^C values in ethane in the two cultures (controls δ-^13^C-DIC, −35‰) with ^13^C-DIC-amended culture with δ-^13^C-DIC = +25,994‰. Based on simple mass balance calculations on the development of fractions, we infer that sulfate-dependent anaerobic AOM in these enrichments is accompanied by a backflow of inorganic carbon amounting to 1 to 3% of the forward rate. This back-reaction indicates a general reversibility of ethane oxidation. Download FIG S4, EPS file, 0.5 MB. Copyright © 2020 Hahn et al. 2020 Hahn et al. This content is distributed under the terms of the Creative Commons Attribution 4.0 International license.

## MATERIALS AND METHODS

### Inoculum and establishment of alkane-oxidizing cultures.

This study is based on samples collected during R/V *Atlantis* cruise AT37-06 with submersible *Alvin* to the Guaymas Basin vent area in December 2016 (for locations, see Table S4 in the supplemental material). A sediment sample was collected by push coring within a hydrothermal area marked by conspicuous orange-type *Beggiatoa* mats (dive 4869, core 26, 27′0.4505′N 111°24.5389′W, 2,001-m water depth, 20 December 2016). The sampling site was located in the hydrothermal area where, during a previous *Alvin* visit, sediment cores containing locally ^13^C-enriched ethane had indicated ethane-oxidizing microbial activity (33). *In situ* temperature measurements using the *Alvin* heat flow probe revealed a steep temperature gradient reaching 80°C at 30- to 40-cm sediment depth. The retrieved samples contained large amounts of natural gas as observed by bubble formation. Soon after recovery, the overlying *Beggiatoa* mat was removed, and the top 10 cm of the sediment was filled into 250-ml Duran bottles, which were gastight sealed with butyl rubber stoppers. In the home laboratory, sediments were transferred into an anoxic chamber. There, a sediment slurry (20% sediment and 80% medium) was produced with synthetic sulfate reducer (SR) medium (pH 7.0) (38, 39) and distributed into replicate bottles (sediment dry weight per bottle, 1.45 g). These bottles were amended with methane or ethane (0.2 MPa) or kept with an N_2_ atmosphere without alkane substrate. These samples were incubated at 37°C, 50°C, and 70°C. To determine substrate-dependent sulfide production rates, sulfide concentrations were measured every 2 to 4 weeks using a copper sulfate assay (40). Ethane-dependent sulfide production was observed at 37°C and 50°C but not at 70°C. When the sulfide concentration exceeded 15 mM, the cultures were diluted (1:3) in SR medium and resupplied with ethane. Repeated dilutions led to virtually sediment-free, highly active cultures within 18 months. A slight decrease of the initial pH value to 6.5 led to increased ethane oxidation activity and faster growth in the culture.

10.1128/mBio.00600-20.8 TABLE S4 Overview of environmental sampling sites used for this study. Download Table S4, XLSX file, 0.02 MB. Copyright © 2020 Hahn et al. 2020 Hahn et al. This content is distributed under the terms of the Creative Commons Attribution 4.0 International license.

### Quantitative substrate turnover experiment.

The Ethane50 culture was equally distributed in six 150-ml serum flasks using 20 ml inoculum and 80 ml medium. Three replicate cultures were amended with 0.05-MPa ethane in 0.1-MPa N_2_-CO_2_, while 3 negative controls were amended with 0.15-MPa N_2_-CO_2_. Both treatments were incubated at 50°C. Weekly, 0.5-ml headspace gas samples were analyzed for ethane content using an Agilent 6890 gas chromatograph in splitless mode equipped with a packed column (Supelco Porapak Q, 6 ft by 1/8 ft by 2.1-mm stainless steel column, oven temperature 80°C). The carrier gas was helium (20 ml per minute), and hydrocarbons were detected by flame ionization detection. Each sample was analyzed in triplicates and quantified against ethane standards of 5, 10, and 100%. Derived concentrations were converted into molar amounts by taking the headspace size, pressure, and temperature into account. Results were corrected for sampled volumes. Sulfide concentrations were measured as described above. To determine sulfate concentrations, 1 ml of sample was fixed in 0.5 ml zinc acetate. Samples were diluted 1:50 with deionized water (MilliQ grade; >18.5 MΩ), and samples were measured using nonsuppressed ion chromatography (Metrohm 930 Compact IC Metrosep A PCC HC/4.0 preconcentration and Metrosep A Supp 5-150/4.0 chromatography column).

### DNA extraction, 16S rRNA gene amplification, and tag sequencing.

DNA was extracted from the different cultures and the original sediment with the Mo Bio Power soil DNA extraction kit (Mo Bio Laboratories Inc., Carlsbad, CA, USA) using a modified protocol. Twenty milliliters of the culture was pelleted via centrifugation (5,000 × *g*; 10 min). The pellet was resuspended in phosphate-buffered saline (PBS) and transferred to the PowerBeat tube (Mo Bio Power soil DNA extraction kit; Mo Bio Laboratories Inc., Carlsbad, CA, USA). The cells were lysed by three cycles of freezing in liquid nitrogen (20 s) and thawing (5 min at 60°C). After cooling down to room temperature, 10 μl of proteinase K (20 mg ml^−1^) was added and incubated for 30 min at 55°C. Subsequently, 60 μl of solution C1 (contains SDS) was added, and the tubes were briefly centrifuged. The samples were homogenized 2 times for 30 s at 6.0 m/s using a FastPrep-24 instrument (MP Biomedicals, Eschwege, Germany). In between the runs, the samples were kept on ice for 5 min. After these steps, the protocol was followed further according to the manufacturer’s recommendations. DNA concentrations were measured using a Qubit 2.0 instrument (Invitrogen, Carlsbad, CA, USA). Two nanograms of DNA was used for amplicon PCR, and the product was used for 16S rRNA gene amplicon library preparation according to the 16S metagenomic sequencing library preparation guide provided by Illumina. The Arch349F-Arch915R primer pair was used to amplify the archaeal V3-V5 region, and the Bact341F-Bact785R primer pair was used for the bacterial V3-V4 region (see Table S1 in the supplemental material). Amplicon libraries for both *Archaea* and *Bacteria* were sequenced on an Illumina MiSeq instrument (2- by 300-bp paired-end run, v3 chemistry) at CeBiTec (Bielefeld, Germany). After analysis, adapters and primer sequences were clipped from the retrieved sequences using cutadapt (41) (v1.16) with 0.16 (−e) as maximum allowed error rate and no indels allowed. Resulting reads were analyzed using the SILVAngs pipeline using the default parameters (https://ngs.arb-silva.de/silvangs/) (42–44).

### Extraction of high-quality DNA, library preparation, and sequencing of gDNA.

Biomass from 200 ml of the Ethane50 and Ethane37 cultures was pelleted by centrifugation and resuspended in 450 μl of extraction buffer. Genomic DNA was retrieved based on a modified version of the protocol described in reference 45, including three extraction steps. Resuspended pellet was frozen in liquid N_2_ and thawed in a water bath at 65°C. Another 1,350 μl of extraction buffer was added. Cells were digested enzymatically by proteinase K (addition of 60 μl of 20 mg/ml, incubation at 37°C for 1.5 h under constant shaking at 225 rpm) and chemically lysed (addition of 300 μl 20% SDS for 2 h at 65°C). Samples were centrifuged (20 min, 13,000 × *g*), and the clear supernatant was transferred to a new tube. Two milliliters of chloroform-isoamyl alcohol (16:1, vol/vol) was added to the extract, mixed by inverting, and centrifuged for 20 min at 13,000 × *g*. The aqueous phase was transferred to a new tube, mixed with 0.6 volumes of isopropanol, and stored overnight at −20°C for DNA precipitation. The DNA was redissolved in water at 65°C for 5 min and then centrifuged for 40 min at 13,000 × *g*. The supernatant was removed, and the pellet was washed with ice-cold ethanol (80%) and subjected to centrifugation for 10 min at 13,000 × *g*. The ethanol was removed, and the dried pellet was resuspended in PCR-grade water. This procedure yielded 114 μg and 145 μg high-quality genomic DNA (gDNA) from the Ethane37 and the Ethane50 cultures, respectively. Samples were sequenced with Pacific Biosciences Sequel as a long amplicon (4 to 10 kb) and long-read gDNA library at the Max Planck-Genome-Centre (Cologne, Germany). To evaluate the microbial community, we extracted 16S rRNA gene reads using Metaxa2 (46) and taxonomically classified them using the SILVA ACT online service (47). For assembly, either HGAP4 (implemented in the SMRTlink software by PacBio) or Canu (https://github.com/marbl/canu) was used. The closed GoM-Arc1 genome from the Ethane37 culture was prepared manually by the combination of assemblies from the two above-mentioned tools. The final genome was polished using the resequencing tool included in the SMRTLink software by PacBio. For noncircularized *de novo* genomes, the resulting contigs were mapped via minimap2 (https://github.com/lh3/minimap2; parameter: ‘-x asm10’) to a reference genome. The reference consensus genomes were prepared using the resequencing tool implemented in the SMRTLink software of PacBio using either the circular GoM-Arc1 *de novo* genome from this study or the publicly available “*Ca.* Desulfofervidus” genome (accession no. NZ_CP013015.1) as reference. Final genomes were automatically annotated using Prokka (48), and the annotation was refined manually using the NCBI BLAST interface (49). Average nucleotide and amino acid identities were calculated using Enveomics tools (50).

### Single-cell genomics.

Anoxic sediment aliquots were shipped to the Bigelow Laboratory Single Cell Genomics Center (SCGC; https://scgc.bigelow.org). Cells were separated, sorted, and lysed, and total DNA was amplified by multiple displacement amplification. Single-cell DNA was characterized by 16S rRNA gene tag sequences (12, 51). The single-cell amplified DNA from Gulf of Mexico samples was analyzed and sequenced as described before in reference 12. Single-cell amplified DNA from Amon Mud Volcano AAA-792_C10 was sequenced with HiSeq 3000 and MiSeq technology, and reads were assembled using SPAdes (52) with the single-cell mode. Assembled reads were binned based on tetranucleotides, coverage, and taxonomy using MetaWatt (53). The final SAG was evaluated for completeness and contamination using CheckM (54). Genome annotation was performed as described above.

### Extraction of RNA, reverse transcription, sequencing, and read processing.

Extraction and sequencing of total RNA was performed in triplicates. RNA was extracted from 150-ml active Ethane50 culture grown in separate bottles at 50°C. Total RNA was extracted and purified as described in reference 9 using the Quick-RNA miniprep kit (Zymo Research, Irvine, CA, USA) and RNeasy MinElute cleanup kit (Qiagen, Hilden, Germany). Per sample, at least 150 ng of high-quality RNA was obtained. The RNA library was prepared with the TruSeq stranded total RNA kit (Illumina). An rRNA depletion step was omitted. The samples were sequenced on an Illumina NextSeq with v2 chemistry and 1- by 150-bp read length. The sequencing produced ∼50-Gb reads per sample. Adapters and contaminant sequences were removed, and reads were quality trimmed to Q10 using bbduk v36.49 from the BBMAP package. For phylogenetic analysis of the active community, 16S rRNA reads were recruited and classified based on SSU SILVA release 132 (47) using phyloFlash (55). Trimmed reads were mapped to the closed genomes of “*Candidatus* Ethanoperedens thermophilum” and “*Ca.* Desulfofervidus” using Geneious Prime 2019.2.1 (Biomatters, Ltd., Auckland, New Zealand) with a minimum mapping quality of 30%. The expression level of each gene was quantified by counting the number of unambiguously mapped reads per gene using Geneious. To consider gene length, read counts were converted to reads per kilobase per million mapped reads (RPKM).

### Phylogenetic analysis of 16S rRNA genes, marker genes, and *mcr*A amino acid sequences.

A 16S rRNA gene-based phylogenetic tree was calculated using publicly available 16S rRNA sequences from the SSU Ref NR 128 SILVA database (42). The tree was constructed using ARB (56) and the FastTree 2 package (57) using a 50% similarity filter. Sequence length for all 16S rRNA genes was at least 1,100 bp. After tree calculation, partial sequences retrieved from single cells were included into the tree. ARB (56) was used for visualization of the final tree. The marker gene tree was calculated using 126 publicly available genomes and genomes presented in this study. The tree was calculated based on aligned amino acid sequences of 32 marker genes picked from known archaeal marker genes (Table S5) (58). For the preparation of the aligned marker gene amino acid sequences, we used the phylogenomic workflow of Anvi’o 5.5 (59). The marker gene phylogeny was calculated using RAxML version 8.2.10 (60) with the PROTGAMMAAUTO model and LG likelihood amino acid substitution. One thousand fast bootstraps were calculated to find the optimal tree according to RAxML convergence criteria. The software iTOL v3 was used for tree visualization (61). The *mcr*A amino acid phylogenetic tree was calculated using 358 sequences that are publicly available or presented in this study. The sequences were manually aligned using the Geneious Prime 2019.2.1 (Biomatters, Ltd., Auckland, New Zealand) interface, and 1,060 amino acid positions were considered. The aligned sequences were masked using Zorro (https://sourceforge.net/projects/probmask/), and a phylogenetic tree was calculated using RAxML version 8.2.10 (60) using the PROTGAMMAAUTO model and LG likelihood amino acid substitution. One thousand fast bootstraps were calculated. The tree was visualized with iTOL v3 (61).

10.1128/mBio.00600-20.9 TABLE S5 Marker genes used for calculation of genome tree based on archaeal marker genes presented in the work of Rinke et al. (58). Download Table S5, XLSX file, 0.01 MB. Copyright © 2020 Hahn et al. 2020 Hahn et al. This content is distributed under the terms of the Creative Commons Attribution 4.0 International license.

10.1128/mBio.00600-20.10 TABLE S6 Summary data set of the development of substrates and products in the Ethane50 enrichment culture. Development of ethane, sulfide, and sulfate concentrations in Ethane50 culture in triplicates. Development of sulfide concentration in Ethane50 culture in 10 replicates. Development of ethane and sulfide concentrations in triplicates of the Ethane50 culture with hydrogen gas (1.5 atm) with and without sulfate. The positive control contained 150 kPa ethane and sulfate. Download Table S6, XLSX file, 0.02 MB. Copyright © 2020 Hahn et al. 2020 Hahn et al. This content is distributed under the terms of the Creative Commons Attribution 4.0 International license.

### Catalyzed reported deposition fluorescence *in situ* hybridization (CARD-FISH).

Aliquots of the Ethane50 culture and environmental samples were fixed for 1 h in 2% formaldehyde, washed three times in PBS (pH 7.4)-ethanol (1:1), and stored in this solution. Aliquots were sonicated (30 s; 20% power; 20% cycle; Sonoplus HD70; Bandelin) and filtered on GTTP polycarbonate filters (0.2-μm pore size; Millipore, Darmstadt, Germany). CARD-FISH was performed according to reference 62 including the following modifications. Cells were permeabilized with a lysozyme solution (PBS [pH 7.4], 0.005 M EDTA [pH 8.0], 0.02 M Tris-HCl [pH 8.0], 10 mg ml^−1^ lysozyme; Sigma-Aldrich) at 37°C for 60 min followed by proteinase K solution treatment (7.5 μg ml^−1^ proteinase K [Merck, Darmstadt, Germany] in PBS [pH 7.4], 0.005 M EDTA [pH 8.0], 0.02 M Tris-HCl [pH 8.0]) at room temperature for 5 min. Endogenous peroxidases were inactivated by incubation in a solution of 0.15% H_2_O_2_ in methanol for 30 min at room temperature. Horseradish peroxidase (HRP)-labeled probes were purchased from Biomers.net (Ulm, Germany). Tyramides were labeled with Alexa Fluor 594 or Alexa Fluor 488. All probes were applied as listed in Table S1. For double hybridization, the peroxidases from the first hybridization were inactivated in 0.15% H_2_O_2_ in methanol for 30 min at room temperature. Finally, the filters were counterstained with DAPI (4′,6′-diamino-2-phenylindole) and analyzed by epifluorescence microscopy (Axiophot II imaging; Zeiss, Germany). Selected filters were analyzed by confocal laser scanning microscopy (LSM 780; Zeiss, Germany) including the Airyscan technology.

### Synthesis of authentic standards for metabolites.

To produce alkyl-CoM standards, 1 g of coenzyme M was dissolved in 40 ml 30% (vol/vol) ammonium hydroxide solution, and to this solution 1.8 to 2 g of bromoethane, bromopropane, or bromobutane was added. The mixture was incubated for 5 h at room temperature under vigorous shaking and then acidified to pH 1 with HCl. The produced standard had a concentration of approximately 25 mg ml^−1^, which for mass spectrometry measurements was diluted to 10 μg ml^−1^.

### Extraction of metabolites from the Ethane50 culture.

In the anoxic chamber, 20 ml of Ethane50 culture was harvested into 50-ml centrifuge tubes. Tubes were centrifuged at 3,000 relative centrifugal force (rcf) for 10 min, and the supernatant was removed. The pellet was resuspended in 1 ml acetonitrile-methanol-water (4:4:2, vol/vol/vol) mixture in lysing matrix tubes (MP Biomedicals, Eschwege, Germany) with glass beads. Afterward, the tubes were removed from the anoxic chamber and the samples were mechanically lysed in a FastPrep homogenizer (MP Bio) with 5 cycles with 6 M/s for 50 s and cooling on ice for 5 min between the homogenization steps. Finally, the samples were centrifuged for 5 min at 13,000 × *g*, and the supernatant was transferred to a new tube and stored at −20°C.

### Solvents for LC-MS/MS.

All organic solvents were liquid chromatography-mass spectrometry (LC-MS) grade, using acetonitrile (ACN; BioSolve, Valkenswaard, The Netherlands), isopropanol (IPA; BioSolve, Valkenswaard, The Netherlands), and formic acid (FA; BioSolve, Valkenswaard, The Netherlands). Water was deionized by using the Astacus MembraPure system (MembraPure GmbH, Berlin, Germany).

### High-resolution LC-MS/MS.

The analysis was performed using a QExactive Plus Orbitrap (Thermo Fisher Scientific) equipped with a heated electrospray ionization (HESI) probe and a Vanquish Horizon ultra-high-performance liquid chromatography (UHPLC) system (Thermo Fisher Scientific). The metabolites from cell extracts were separated on an Accucore C_30_ column (150 by 2.1 mm, 2.6 μm; Thermo Fisher Scientific), at 40°C, using a solvent gradient created from the mixture of buffer A (5% acetonitrile in water, 0.1% formic acid) and buffer B (90/10 IPA-ACN, 0.1% formic acid). The solvent gradient was the following: fraction B of 0, 0, 16, 45, 52, 58, 66, 70, 75, 97, 97.15, and 0%, at −2 min (prerun equilibration) and 0, 2, 5.5, 9, 12, 14, 16, 18, 22, 25, 32.5, 33, 34.4, and 36 min of each run, and a constant flow rate of 350 μl min^−1^. The sample injection volume was 10 μl. The MS measurements were acquired in negative mode for a mass detection range of 70 to 1,000 Da. In alternation, a full MS and MS/MS scans of the eight most abundant precursor ions were acquired in negative mode. Dynamic exclusion was enabled for 30 s. The settings for full-range MS1 were mass resolution of 70,000 at 200 *m/z*, automatic gain control (AGC) target of 5 × 10^5^, and injection time of 65 ms. Each MS1 was followed by MS2 scans with the following settings: mass resolution of 35,000 at 200 *m/z*, AGC target of 1 × 10^6^, injection time of 75 ms, loop count of 8, isolation window of 1 Da, and collision energy set to 30 eV.

### Determination of carbon back flux into the ethane pool.

Aliquots of active AOM culture (50 ml) were transferred into 70-ml serum bottles with N_2_:CO_2_ headspace. In the stable-isotope probing (SIP) experiment, addition of 99% ^13^C-labeled dissolved inorganic carbon (DIC) (1 ml, 350 mM) led to δ-^13^C-DIC values of +25,000‰ as measured by cavity ringdown spectrometry. Ethane (2 atm = 1.8 mM) was added to both experiments, and cultures were stored at 50°C. To determine the overall ethane oxidation activity, sulfide concentrations were measured every few days as described above and converted to ethane oxidation rates using ratios in the chemical formula in Results and Discussion. To measure the development of ethane δ-^13^C values, 1 ml of the gas phase was sampled every few days and stored in 10-ml Exetainer vials with 2 ml NaOH, and ethane isotopic composition was measured using gas chromatography coupled via a combustion interface to isotope ratio mass spectrometry (Trace GC Ultra with Carboxene-1006 Plot column, 40°C oven temp., carrier gas He with flow rate 3 ml min^−1^; coupled via GC IsoLink to Delta V isotope ratio MS).

### Net ethane production test.

To test for net ethane production, in 156-ml serum flasks replicate incubations with about 0.5 g (wet weight) active Ethane50 culture in 100 ml of sulfate-free medium was prepared. Four different conditions were tested in three biological replicates with the addition of (i) 1.5 atm H_2_; (ii) conditions replicating the first but with only 0.05 g biomass; (iii) 1.5 atm H_2_ plus 28 mM sulfate; and (iv) an activity control with addition of sulfate and 1.5 atm ethane. Cultures were incubated over 27 days at 50°C, and sulfate and ethane concentrations were monitored as described above.

### Data availability.

All sequence data are archived in the ENA database under the INSDC accession numbers PRJEB36446 and PRJEB36096. Sequence data from Loki’s Castle are archived under NCBI BioSample number SAMN13220465. The 16S rRNA gene amplicon reads have been submitted to the NCBI Sequence Read Archive (SRA) database under the accession number SRR8089822. All sequence information has been submitted using the data brokerage service of the German Federation for Biological Data (GFBio) (63), in compliance with the Minimal Information about any (X) Sequence (MIxS) standard (64), but some data are still under ENA embargo.
